# Effectiveness of nurse-led hypertension interventions on retention, adherence, and blood pressure control among adults in low- and middle-income countries: A systematic review

**DOI:** 10.1371/journal.pone.0362190

**Published:** 2026-11-05

**Authors:** Tagese Yakob, Charuai Suwanbamrung, Cua Ngoc Le, Abebe Sorsa Badacho, Udomsak Saengow

**Affiliations:** 1 Public Health Research, School of Public Health, Walailak University, Nakhon Si Thammarat, Thailand; 2 Excellent Center for Public Health Research: EC for PHR, Walailak University, Nakhon Si Thammarat, Thailand; 3 South Ethiopia Region Public Health Institute, Jinka, Southern Regional State, Ethiopia; 4 School of Public Health, College of Health Science and Medicine, Wolaita Sodo University, Wolaita Sodo, Ethiopia; 5 Division of Health Systems and Public Health, Department of Global Health, Faculty of Medicine and Health Science, Stellenbosch University, Cape Town, South Africa; 6 Center of Excellence in Data Science for Health Study, Walailak University, Nakhon Si Thammarat, Thailand; 7 School of Medicine, Walailak University, Nakhon Si Thammarat, Thailand; University of Oulu, FINLAND

## Abstract

**Background:**

Hypertension affects more than 1.28 billion adults globally and remains the leading modifiable risk factor for cardiovascular disease, stroke, renal failure, and premature death. This burden is inconsistently distributed across Low- and Middle-Income Countries (LMICs), where nearly three-quarters of adults live with hypertension. This gap is largely driven by poor adherence and low retention. This systematic review aims to assess the effectiveness of nurse-led interventions on retention in care, medication adherence, and blood pressure among adult patients with hypertension in primary health care settings in LMICs.

**Methods:**

The protocol was registered (PROSPERO CRD420251136515) following the PRISMA guidelines. We searched PubMed, Scopus, and Cochrane for articles published between 2000 and 2026. Eligibility criteria included: adults (≥18 years) with hypertension receiving care from primary care clinics; nurse-led interventions; usual care as comparator; primary outcome: retention rates; secondary outcomes: medication adherence, blood pressure (BP) control. Inclusion: experimental, quasi-experimental, and prospective cohort studies. Dual independent screening and data extraction were performed. Quality assessment was performed using Cochrane Risk of Bias 2(RoB2) for randomized controlled trials and Joanna Briggs Institute (JBI) tools for quasi-experimental and observational studies. Due to heterogeneity in intervention duration, outcome measures, and follow-up periods, a narrative synthesis was employed.

**Results:**

Eleven unique studies (*n* = 19,005) were identified: 63.6% RCTs, 18.2% quasi-experimental, and 18.2% observational. Seven studies were conducted in Africa and four in Asia. The mean age range was 52.0–67.4 years; 51.3%−76.0% were female. Risk-of-bias assessment revealed high or unclear risk in the missing outcomes data domain for most RCTs. Ten studies used multicomponent interventions. Retention rates varied with appointment attendance (12.6–96.5%), routine clinical retention (27.0–62.8%), and study completion of follow-up (83.8% to 97.2%). Four studies reported medication adherence with varying effects in the intervention group compared to the control or baseline. Ten studies reported systolic blood pressure (SBP) outcomes; 9 reported mean SBP after intervention; the majority reported the mean reduction of SBP was greater than 10 mmHg. Nine studies reported diastolic blood pressure (DBP) outcomes; all studies reported a reduction in DBP after the intervention, with a range of −1.8 mmHg to −20.6 mmHg. Reported SBP and DBP reductions included both within-group controlled estimates; these should not be interpreted as a pooled effect.

**Conclusion:**

This systematic review included 11 studies that showed nurse-led interventions improved blood pressure control in primary health care facilities of LMICs. But retention in care showed substantial variability in nurse-led interventions in primary health care facilities of LMICs. Nurse-led interventions in LMICs are a promising strategy for improving blood pressure control. However, retention in care was substantially and variably due to heterogeneous definitions and evidence on medication adherence was limited. Future research should prioritize standardized outcome measures and robust study designs to strengthen the evidence base and inform sustainable scale-up of these models in LMICs.

## Background

Hypertension is a major global public health concern and affects more than 1.28 billion adults globally [1]. It remains the leading modifiable risk factor for cardiovascular disease, stroke, renal failure, and premature death [2–4]. This burden is inconsistently distributed across low- and middle-income countries (LMICs) [5]. Despite improved diagnosis and treatment coverage, less than one in five people with hypertension achieves effective control. Two interrelated implementation challenges largely drive this gap: long-term poor adherence and low retention in treatment programs [6–8].

While clinical interventions across LMICs have demonstrated improvements in blood pressure control, they remain insufficient without structured strategies specifically designed to promote treatment retention and medication adherence. Studies conducted across LMIC health care settings revealed that greater than half of patients were lost to follow-up [6,9,10]. Evidence from other LMICs indicates that even effective interventions face retention challenges. For instance, community-based programs in Ghana reported a 25% default rate, and an implementation program conducted in Nigeria reported a 33.3% [11] default rate, highlighting that without deliberate retention strategies, clinical improvement is ineffective [12].

Given these systemic challenges, task-shifting to nurses emerged as a promising approach to strengthen continuity in chronic care in resource-limited settings. In contrast to general task-shifting or sharing to other mid-level health workers, nurse-led primary care coordinators deliver structured behavioral interventions, including health education and counseling on lifestyle modification and self-management, and treatment monitoring. Nurses serve as the most consistent point of contact for patients within often resource-limited healthcare systems [13]. Their continued presence facilitates the longitudinal follow-up and consistent advice, counselling, and health education required for effective chronic non-communicable disease management [13]. It can bridge services faster and cost-effectively [14]. Evidence from LMIC settings suggests that nurse-delivered behavioral interventions reduce SBP and DBP [15]. Task-shifting to nurses for hypertension care is feasible in resource-limited settings, which improves key processes and outcomes when adequately supported [16].

Previous reviews focused on allied health professional-led intervention for hypertension, primarily synthesizing clinical outcomes such as BP control [17–19]. However, patient retention in care and medication adherence were overlooked as process outcomes that are essential to sustaining hypertension management. Documented evidence indicates that improvements in blood pressure depend primarily on patients remaining in care and adhering to prescribed drug regimens. High loss to follow-up, as reported in implementation studies (Ghana: 25% and Ethiopia: 33%), indirectly reflects adherence challenges [20]. In the current context, intervention for hypertension by nurse-led care may be helpful, including increased contact time with patients to improve patient retention in care, medication adherence, and blood pressure control over usual care, improve quality of care, and enhance the self-efficacy of patients to improve control of blood pressure [17].

Existing systematic reviews primarily focused on nurse-led BP control [17,21,22]. Furthermore, reviews evaluated nurse-led hypertension interventions restricted to the clinical endpoint (blood pressure control) outcomes; not restricted to low- and middle-income countries, and included studies from both high-income countries and restricted to controlled studies [23,24], while the current study is different by being restricted to a primary health care setting in LMICs, evaluated three interconnected outcomes: retention, medication adherence, and BP control, and the study is not restricted to controlled studies; rather included uncontrolled observational and implementation studies to evaluate real-world applicability and the studies assessed how intervention duration influences outcomes across them. This systematic review aims to evaluate the effectiveness of nurse-led interventions on retention in care as the primary outcome and medication adherence and blood pressure as secondary outcomes, among adult patients with hypertension in primary health care settings in low- and middle-income countries (LMICs). The findings will inform the design and scale-up of the differentiated nurse-led hypertension interventions. Moreover, the timeframe within which patients achieve meaningful retention, medication adherence, and clinical BP control has not been established. Importantly, retention in care is one of the five themes identified in the WHO’s 2024 research priority areas of hypertension care delivery [25].

## Methods and materials

### Study design

The review protocol was prospectively registered (PROSPERO CRD420251136515). The structured design, execution, and reporting of this review strictly follow the Preferred Reporting of Systematic Reviews and Meta-Analyses (PRISMA) 2020 guidelines (S1 File, Fig 1).

**Fig 1:**
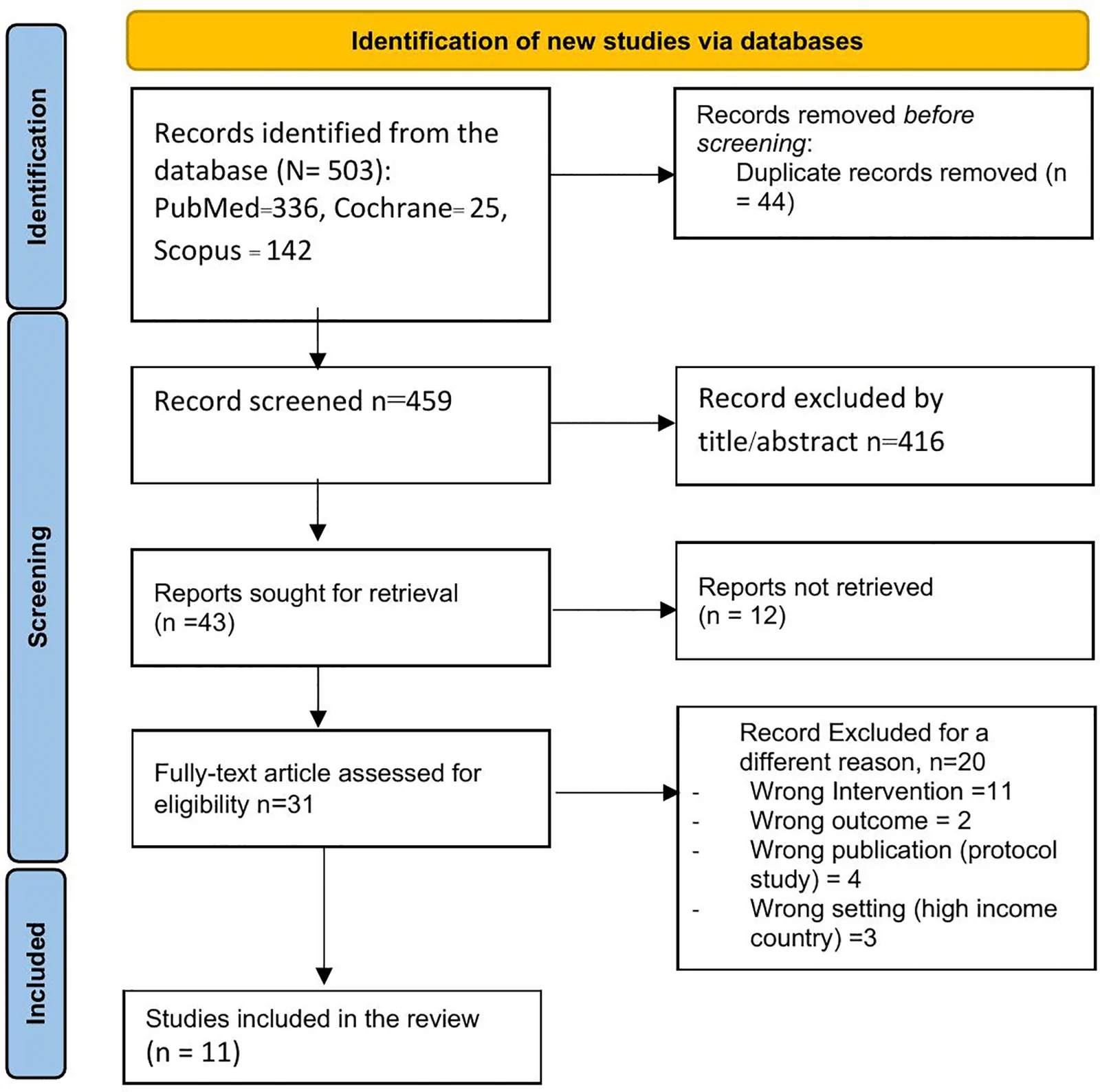
PRISMA Flow Diagram for study selection.

### Eligibility criteria

The current review employed the following population, intervention, comparator, outcomes (PICOS) framework:

**Population:** Studies that included adults (age ≥ 18 years) with hypertension and patients who were selected or enrolled in the study from primary care clinics.

**Intervention:** For the current review, a nurse-led intervention is operationally defined as any structured hypertension care model delivered by a trained nurse (or nurse practitioner/advanced practice nurse) who holds primary responsibility and clinical decision-making for at least three of the following: 1) blood pressure measurement and monitoring, 2) initiation and adjustment of antihypertensive drug treatment according to standard treatment protocol, 3) structured patient education and counselling on medication adherence and lifestyle modification, 4) scheduling and tracking of follow-up visits, and 5) coordination with community health worker or digital monitoring system for continuity of care. Studies included regardless of whether nurses play a role within task-shifting or task-sharing, provided that the nurse played a central rather than supplementary or administrative role. Eligible settings include primary hospitals, health centers, community health centers, and community health clinics in LMICs.

**Comparator:** standard or usual care, minimal intervention, or single-arm pre-post intervention with no control group. Uncontrolled pre-post and implementation studies were eligible to capture real-world retention data in settings where RCTs were not feasible.

**Outcomes:** The primary outcome was retention in care (e.g., attending clinical appointments, continuity in care). Secondary outcomes were medication adherence and blood pressure control rates.

**Study design:** randomized controlled trial, non-randomized controlled trails, single-arm quasi-experimental (pre-post), and observational studies were included

### Duration of intervention

One challenge for administrators, researchers, and clinicians who want to systematically evaluate hypertension clinical engagement is determining how to measure retention in care. Measuring retention is complex because it includes multiple clinic visits scheduled at varying intervals and occurring longitudinally. Due to heterogeneity of retention outcomes across the study, we organized them into three mutually exclusive categories based on underlying clinical or research mechanism. Routine clinical retention category (based on routine visits per expected time period, for example, ≥ 6 visits per year), appointment attendance or provider contact (based on typical follow-up time periods or provider contact on specific recommended period: 1–3 months, 3–6 months, 6–12 months, and more than 12 months), and study completion of follow-up.

### Information sources

Following prospective protocol registration (CRD420251136515), we searched multiple electronic databases (PubMed, Scopus, and Cochrane Central Register of Controlled Trials) for articles published in English between 2000 and February 2026. The 2000 start date was prespecified in the PROSPERO protocol before data searching, and ending boundary was February 2026, when the final literature search was updated; this was based on the policy framework alignment, as non-communicable disease control and prevention was inaugurated in 2000 [26] and a study conducted before 2000 showed that observed interventions before 2000 were solely in clinical treatment algorithms and evidence limited transferability to modern primary health care [27]. Furthermore, given recent advances in information and communication technology that can influence retention, we restricted the study period to the last two and a half decades to make contextualization and adaptation of interventions to current primary health care settings more relevant and applicable [28,29].

### Search strategy

We searched three databases based on a comprehensive search approach. The search strategy incorporated keywords and controlled vocabulary terms for nurse involvement, hypertension, primary care, retention, medication adherence, and blood pressure control. We developed the search strategy in PubMed, Cochrane, and Scopus databases (S2 File).

### Study selection

We conducted a pilot screening to calibrate reviewers and improve screening reliability. Reviewers applied the eligibility criteria to independently screen. We resolved discrepancies through discussion, including with a third author. We repeated this exercise in subsequent pilot screenings until we reached 90% agreement. These same two authors then utilized Covidence to independently screen the titles and abstracts of the remaining potentially relevant articles in duplicate (level 1 screening). Two reviewers then independently reviewed full-text articles to assess eligibility in duplicate (level 2 screening). Disagreements were resolved through discussion, including with a third author, to achieve consensus at each level of screening. Final agreement was reached on 11 unique studies that fulfilled the inclusion criteria for the final data synthesis stage

### Data extraction

We developed a standardized data abstraction form, which was pilot-tested with 2 included studies to ensure agreement among data extractors. Data from included articles were independently extracted and mapped by two reviewers into key domains, including basic characteristics, first authors, country, years, study design, study setting, sample size, intervention timing and frequency, comparators, duration of intervention, primary outcome, secondary outcome, and methods of follow-up. Details of retention interventions included type, timing, and frequency.

Because some interventions to improve retention were multifaceted with multiple targets, we extracted information about each component from all trials, explored its elements, and determined the overall impact through a narrative synthesis. To ensure the synthesis was rigorous and transparent, review authors met to discuss and categorize different retention interventions from the included studies. The independent results were discussed, and discrepancies were reconciled before providing the final list of major retention categories.

### Quality assessment of included studies

Quality assessment of included studies was performed using the Cochrane Risk of Bias 2(RoB2) Tool for RCTs. It was evaluated across five domains: bias raised by randomization, bias due to deviation from the intended intervention, bias due to missing outcome, bias due to measurement of the outcome, and bias due to the selection of the reported result. Overall risk of bias was judged as low risk, some concern, or high risk based on the Cochrane Risk of Bias 2 guide [30]. For both quasi-experimental and observational studies, the Joanna Briggs Institute (JBI) critical appraisal tools were employed, and they had 7 components of assessment for risk assessment of quasi-experimental and 11 components of assessment for observational studies. Each key component was scored yes/no/not applicable/unsure. Overall risk of bias was categorized as low, moderate, or high based on the proportion of fulfilled criteria (with scores <50 indicating low risk, 50%−70% moderate risk, and ≥70% indicating low risk) (Fig 2a, Fig 2b, Fig 2c, S3 File).

**Fig 2:**
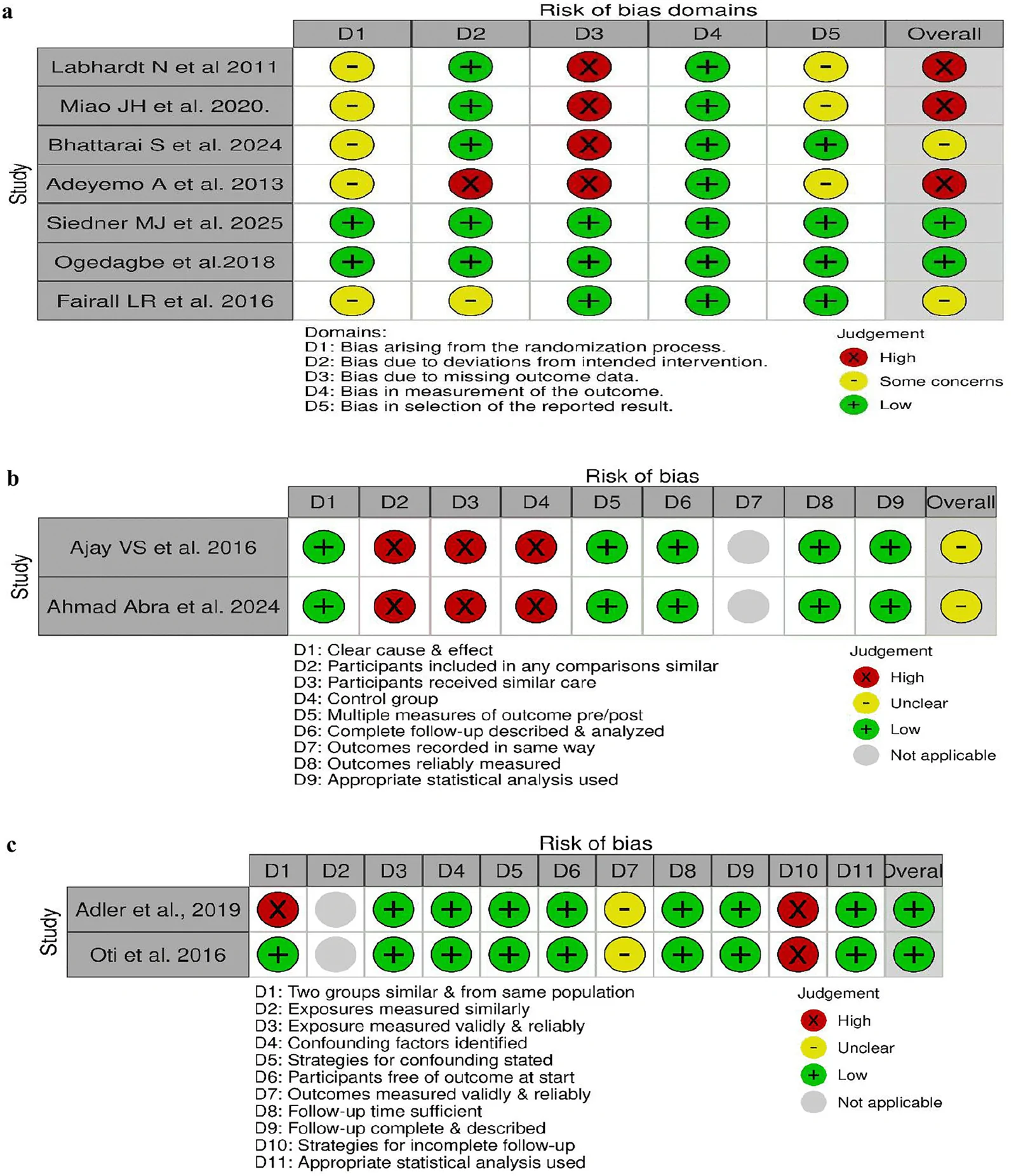
Risk of bias.

### Data synthesis

Due to the heterogeneous study design, measurement outcomes, duration of follow-up, and intervention modality across included studies, the current review did not employ meta-analysis. Instead, findings of the current review were synthesized narratively and current review following the Synthesis Without Meta-analysis guideline (SWIM) [31]. Studies were grouped by retention in care, medication adherence, and blood pressure control (systolic and diastolic). Under each group, the study descriptively narrated the intervention, component, and setting, and implementation contextual facilitators and barriers for retention in care. Effectiveness of the intervention across the outcomes within included studies of the respective outcome was descriptively narrated, adjusted between-group effects, unadjusted between-group effects, intervention-arm pre–post changes, and uncontrolled pre–post changes to distinguish level of effect. The reported median reductions in systolic and diastolic blood pressure across included studies were a descriptive summary of evidence and did not strictly reflect pooled treatment effects.

### Ethical statement

This systematic review used published aggregate data only; no ethical approval was required.

## Results

### Study selection and flow

A total of 503 records were identified across three databases, which were reduced to 459 after the removal of duplicates. Following initial screening of titles and abstracts, 416 were excluded. Of 43 reports sought for retrieval, 12 could not be retrieved, leaving 31 records for full-text articles to be assessed for eligibility. Finally, 11 studies that met eligibility criteria were included in the current review, and 20 studies were excluded from the review for different reasons, including 11 records for wrong intervention, 2 records for wrong outcome, 4 records for wrong publication (protocol study), and 3 records for wrong setting (high-income country) (Fig 1).

### Risk of bias and quality of included studies

Current review quality of data assessment varies across included studies. In the current review, 11 study included; seven were controlled studies and were assessed using the Cochrane RoB2 risk of bias assessment to evaluate controlled studies (Fig 2a, S3 File), 2 studies were quasi-experimental and assessed using JBI tools for quasi-experimental assessment, with 10 key assessment components and scores for each (Fig 2b, S3 File). Two studies were observational and were assessed using the JBI observational study tools (Fig 2c, S3 File).

Among the seven included controlled studies, three studies were categorized as two studies were low risk [32,33], high risk [34–36] and two studies were categorized as having some concern risk of bias [37,38] (Fig 2a, S3 File). Among the two included quasi-experimental studies [39,40] both were between 50–69% moderate risk of bias (Fig 2b, S3 File). Among the two included observational studies [41,42] both were ≥70% low risk of bias (Fig 2c, S3 File).

### Characteristics of the included studies

Eleven studies were included in the review, comprising a total sample size of 19,005 participants (ranging from 156 to 6016). Of the included studies, 7 (63.6%) were randomized controlled trials, 2 were quasi-experimental (18.2%), and 2 were observational (implementation and cohort) studies (18.2%). Of the studies included in the review, 7 were in Africa (2 in Ghana, 2 in South Africa, 1 in Kenya, 1 in Cameroon, and 1 from Nigeria) [32,34,38,41,42], and the others were from Asian countries (China [35], India [39], Nepal [37], and Bangladesh [40]). All studies were conducted in low- and middle-income countries (LMICs) based on the World Bank’s classification [43]. The mean age of participants across the studies ranged from 52.0 to 67.4 years variability of 8–15 years (SD). Among the participants, between 51.3%−76.0% of participants were female (Table 1).

**Table 1:** Presents the characteristics of the included studies.

Authors	Study design	Country	Population mean age, sex	Setting	Number of patients (total sample in study)
**Labhardt ND et al [34]**	Cluster RCT	Cameroon	55 ± 15 years, 57% females	Rural primary care facilities	221(IG = 132, CG = 89)
**Siedner MJ et al [32]**	RCT	South Africa	62 years, 76% of females	Rural primary care clinics	774 (IG = 515, CG = 259)
**Adeyemo A et al [36]**	RCT	Nigeria	63 years, NR	Urban and rural primary clinics	668 (IG = 334, CG = 334)
**Ogedegbe G et al. [33]**	Cluster RCT	Ghana	61 ± 12 years, NR	Community health center	757 (IG = 368, CG = 389)
**Oti SO et al. [42]**	Implementation study	Kenya	>35 years, NR	Urban slum clinic	I = 660
**Miao J-H et al. [35]**	RCT	China	67.4 ± 7.8 years, 51.3% of females	Urban community health center	156 (IG = 78, CG = 78)
**Ajay VS et al. [39]**	Pre-post quasi-experimental	India	55 ± 10 years, sex NR	Community health center	I = 6,016
**Bhattarai S et al [37]**	Cluster RCT	Nepal	57.5 ± 7.8 years, 60% of females	Urban public health facilities	1252 (IG = 627, CG = 625)
**Adler AJ et al [41]**	Prospective cohort	Ghana	58 years, 69% of females	Rural community health clinics	I = 1339
**Fairall LR et al. [38]**	Cluster RCT	South Africa	52 years, 73% of females	public sector primary care clinic	3,227(IG = 1555, CG = 1672)
**Abrar A et al. [40]**	Matched-pair cluster quasi-experimental trial	Bangladesh	Mean 52.3 ± 12.4 years, 70.5 females	Primary healthcare facilities	3935 (IG = 1950, CG = 1985)

**Footnote**: IG: intervention group, CG: control group, I=studies with no control group, NR=not reported

### Characteristics of retention rates in the included studies

Current review evidence demonstrated wide variability of retention across the included studies. Furthermore, different included studies used different retention measurement approaches, and the follow-up period also varied. From the included studies in the current review, two studies evaluated retention outcomes based on routine clinical retention categories [34,42]. Based on this measurement of included studies, retention ranged from 27% at 18 months [42] to 62.8% at 12 months [34]. From the included studies in the current review, five studies evaluated retention outcomes based on measuring appointment attendance or provider contact [32,35,39–41] ranging from 12.6% for long-term follow-up at 18 months [39] to 96.5% at 6 months for single visit completion [40]. Among the included studies in the review, four studies revealed retention outcomes based on completion of follow-up [33,36–38]. In this category, studies revealed retention ranging from 83.8% [38] to 97.2% [36] across the included studies (Table 2).

**Table 2:** Characteristics of retention in care of included studies.

Study	Retention definition	Follow-up duration	Intervention arm, n/N, (%)	Control arm, n/N, (%)	Effect estimate, 95% CI	Status of adjustment
Retention outcome category of routine clinical retention/care continuity						
Labhardt ND et al [34]	≥12 clinic visits within 12 months, and a core outcome linked with the program’s effectiveness	12 months	83/132(62.8%)	26/89(29.2%)	RR = 2.18(1.48–3.22)	Adjusted for cluster
Oti SO et al [42]	≥6 visits within 12 months and an indicator for feasibility and service delivery	18 months	178/660(27%)	N/A	Pre-post	Unadjusted
Retention outcome category of appointment attendance						
Siedner MJ et al [32]	Any provider interaction in the past 3 months	6 months	489/515(94.5%)	255/259(98.5%)	RR = 0.96(0.94–0.99)	Unadjusted
Ajay VS et al. [39]	Attendance at the 18-month visit in the subgroup	18 months	759/6016(12.6%)	N/A	Pre-post	Unadjusted
Adler AJ et al. [41]	Attendance at 12-month visits	12 months	338/1,339(25.2%)	N/A	Cohort completion	Unadjusted
Abrar A et al. [40]	Completion of 6-month follow-up visit	6 months	1882/1950(96.5%)	1876/1985(94.5%)	IRR = 0.22(0.15, 0.32)	Adjusted for clustering
Miao J-H et al. [35]	Completion of a 4-month follow-up visit	4 months	56/78(71.8%)	69/78(88.4%)	RR = 1.23(1.04–1.46)	Unadjusted
Retention outcome category of study follow-up completion						
Ogedegbe G et al. [33]	Completion of 12-month follow-up	12 months	324/368(88.0%)	319/389(82.0%)	aOR=1.61(1.07-2.43)	Unadjusted for cluster
Fairall LR et al. [38]	Completion of a 14-month follow-up visit (subcohort with and without hypertension)	14 months	1,512/1,555(97.2%)	1,626/1,672(97.2%)	NR (calculated RR = 1.00(0.99–1.01))	Unadjusted
Bhattarai S et al [37]	Completion of 10–12 months follow-up, and an Indicator of key program performance	12 months	554/627(88.4%)	544/625(87.0%)	RR = 1.02(0.98–1.05)	Adjusted
Adeyemo A et al [36]	Completion of 6-month follow-up	6 months	280/334(83.8%)	264/334(79.0%)	RR = 1.06(0.99–1.14)	

Footnote: IG: intervention group, CG: control group, I: studies with no control group, N/A = not- applicable, RR = relative risk, IRR = intervention risk ratio, NR = not reported, aOR=adjusted odds ratio.

### Interventions for improving retention in hypertension care

Across the eleven studies, to improve retention, a multicomponent intervention was predominantly used, including multilevel approaches integrating clinical, counseling/educational, digital tracking, and health system components. The majority of studies, 10 out of 11 studies, approached more than two intervention domains; for example, a combination of clinical + education +digital tracking (multicomponent) was utilized. Patient counseling/education, scheduling follow-up adherence visits, and integration of a digital reminder tracking system were the predominant approaches that studies used. Some studies used a standardized protocol [38,40]. However, clinical care alone is supported by digital monitoring systems [39] (Table 3). Even though the intervention component was well described, only a few studies reported and explicitly predefined retention as a process outcome, which shows there is a gap in implementation reporting standards.

**Table 3:** Interventions for improving retention in hypertension care.

Authors	Setting	Key Nurse-led Intervention Components	Primary retention mechanism
**Labhardt ND et al [34]**	Rural primary care facilities	-protocol-driven hypertension clinics -BP measurement at each visit -Treatment according to protocol -Patient counseling during clinic visit -Scheduling and recording follow-up visit	-Structured visit tracking and clinical protocol adherence
**Siedner MJ et al [32]**	Rural primary care clinics	-BP assessment -Ongoing patient contact through the clinic and telemonitoring -Coordination with community health workers -follow-up monitoring	-Tracking digital monitoring
**Adeyemo A et al [36]**	Urban and rural primary clinics	-nurse-managed hypertension clinic -BP measurement -Home visits on the other intervention arm -Medication adherence counseling -Pill counts -Urine tracer monitoring	Multilevel tracking and monitoring approach (clinical, home and biochemical)
**Ogedegbe G et al [33]**	Community health center	-BP assessment -nurse-led hypertension care -lifestyle counseling -medication education -scheduled and arranged follow-up visits	Structured appointment scheduling and patient education
**Oti SO et al [42]**	Urban slum clinic	-Clinic-based hypertension management -BP assessment and monitoring SMS reminders -Close monitoring appointment frequency/ visits	SMS reminders and intensified follow-up frequency
**Miao J-H et al [35]**	Urban community health center	-Structured nurse-led hypertension care -Patient education -Counselling on medication adherence -Close BP assessment and monitoring -Assessing adherence based on score	Retention adherence tracking with counselling
**Ajay VS et al. [39]**	Community health center	-BP assessment -Use of a mobile decision support system -Counseling based on algorithm output -Manage and close monitoring of appointments and follow-up visits	Proactive scheduling and clinical digital support
**Bhattarai S et al [37]**	Urban public health facilities	-BP measurement -Close supervision and monitoring of community health worker home visits -Medication-related support -Follow-up completion monitoring	Task-sharing with follow-up tracking
**Adler AJ et al [41]**	Rural community health clinics	-Follow-up hypertension care -Review of digitally recorded BP data -Education and counselling during the appointment visit -Digital coordination	Counselling and digital tracking
**Fairall LR et al [38]**	Public sector primary care clinic	-Routine hypertension care -BP assessment -Protocol-based hypertension management	Protocol-driven follow-up with the trained nurse
**Abrar A et al [40]**	Rural Upazila Health Complexes (UHCs) – primary healthcare facilities	WHO-HEARTS technical package -Three-step, drug-specific, and dose-specific hypertension treatment protocol -Standardized inventory and procurement practices for 30-day prescriptions -Team-based care (medical officers, nurses, medical assistants) -Systematic approach to follow-up and patient retention -Digital health information system using a simple app	Protocol-driven multisystem redesign +HIS digital tracking

### Contextual barriers and facilitators of retention in care for hypertension

From the 11 studies included in the review, 9 studies [32–34,36,37,39–42] reported the contextual barriers and facilitators of retention in care for hypertension during their intervention window period. The predominant report of barriers was financial/cost-related, with transport or medicines [32–34,39–42], access to the facility [32,33,36,37,39,41,42], patient-level issues like forgetfulness and lack of time for follow-up [34,36,42], and a lack of access to digital support in the health facility [32,39–41], while financial support [34,36,37], digital support [32,34,39–42], task-shifting approach [32–34,36,37,39] and a system of facility improvement, like protocol-driven follow-up of patients [33,40,42] were predominantly reported as facilitators of studies (Table 4).

**Table 4:** Contextual barriers and facilitators identified in retention in care for hypertension included studies.

Authors	Facilitators	Barriers
**Labhardt ND et al [34]**	-Financial incentive related to free medication -Reminder letter -Nurse-patient treatment contract	-Transport cost -Time constraints -Forgetting an appointment -Death, but unrelated to intervention
**Siedner MJ et al [32]**	-Home visits by CHW reduce transport costs -Telemonitoring -Ongoing nurse contact	-Network problem -Distance to facilities -Cost-related
**Adeyemo A et al [36]**	-free medication for care -transport support -Nurse-led follow-up and home visits	-adverse drug effects - Comorbidity (stroke, CHF) -loss of interest in care -Use of alternative medicine -Relocation
**Ogedegbe G et al [33]**	-structured clinical follow-up schedule and nurse-led task shifting	-relocation/migration -Withdrawal from follow-up study -Cost-related
**Oti SO et al [42]**	-Presence of reminder (SMS) -Improved access	-Cost of care -Lack of time -Forgetting appointments -High mobile slum population
**Ajay VS et al [39]**	-Technology support for decision(mDSS) -Appointed person for coordination (nurse)	-Digital platform crash -Cost of care -Distance to clinical care
**Bhattarai S et al [37]**	-Regular home visits by CHW reduce transport-related costs -Appointed a supervisor (nurse) -Provided pill box support for daily medication use	-Relocation of the study population from the study area -Unrelated death with intervention and withdrawal of participant from the study
**Adler AJ et al [41]**	-Use of digital to follow up (CommCare) -Use of SMS reminders helped to recall the appointment	-Digital System failure due to the crash of the digital platform Cost of care -Distance to health care
**Abrar A et al [40]**	-Simplified mDSS version 2.0 with fewer data points (23 vs 36) -High acceptance from Medical Officers and patients	-A high number of data elements in the initial mDSS is choking patient flow -Medicine supply insufficiency at pharmacies (~80% of patients relied on out-of-pocket spending).

### Characteristics of medication adherence

Out of 11 studies included in the current review, 4 studies [35–37,40] reported medication adherence outcomes. Evidence demonstrated substantial variability in medication adherence measurement across the included studies. Such as pill count, Self-reported medication adherence during follow-up, Satisfactory urine assay test, MMAS-8, and an adherence scoring system, reflecting that the included studies used heterogeneous measurements (Table 5).

**Table 5:** Characteristics of medication adherence included in the study.

Authors	Adherence measure	Adherence intervention, n/N, (%)	Adherence control, n/N, (%)	Effect estimate, 95% CI	Intervention types given	Adjustment status	Follow-up duration
**Adeyemo A et al [36]**	Pill count ≤2 excess/ month	203/280(72.5%)	216/264(81.8%)	aOR=0.52(0.34-0.80)	-Nurse-managed hypertension clinic -BP measurement -Home visits on the other intervention arm -Medication adherence counseling -Pill counts -Urine tracer monitoring	adjusted	6 months
	Satisfactory urine assay (≥3 positive)	236/280(84.3%)	220/264(83.3%)	aOR=1.05(0.63-1.73)	-Nurse-managed hypertension clinic -BP measurement -Home visits on the other intervention arm -Medication adherence counseling -Pill counts -Urine tracer monitoring	adjusted	6 months
**Miao J-H et al. [35]**	Adherence scoring system (Time, frequency, dose) (0-3)	Median 3(IQR = 2–3)	Median 3(IQR 2–3)	NR	Structured nurse-led hypertension care -patient education -Counselling on medication adherence -Close BP assessment and monitoring -Assessing adherence based on score	Unadjusted	4 months
**Bhattarai S et al [37]**	MMAS-8 (used score >6)	470/627(77.9%)	394/625(68.3%)	aOR=0.9(0.6-1.5)	-Medication-related support -Follow-up completion monitoring	adjusted	12 months
**Abrar A et al. [40]**	Self-reported medication adherence during follow-up	1,860/1950(95.4%)	1,503/1985(75.7%)	NR(P < 0.001)	WHO-HEARTS technical package	Unadjusted	6 months

MD = mean difference, aOR=adjusted odds ratio, NR = not reported, IQR = interquartile range, MMAS = Morisky Medication Adherence Scale, *endline value calculated from baseline and mean reduction

### Characteristics of blood pressure control

#### Characteristics of the systolic blood pressure (SBP).

Among studies included in the current review, 10 reported SBP outcomes [32–35,37–40,42]. Baseline SBP ranged from 134.0 mmHg [38] to 175.8 mmHg [34] and endline SBP ranged from 129.8 mmHg [37] to 141.6 mmHg [42] among intervention groups, whereas baseline SBP ranged from 133.4 [40] mmHg to 158.8 mmHg [37] and endline SBP ranged from 130.8 mmHg [37] to 144.8 mmHg [32] among the control groups. Out of 10 studies that reported SBP, nine reported [32–35,37,39,40,42] mean SBP reduction, and of them, 8 reported greater than 10 mmHg mean SBP reduction [32–35,39–42] after intervention. But one study reported a mean SBP increase [38] after intervention (Table 6).

**Table 6:** Characteristics of SBP Control of included studies in review.

Authors	Follow-up	Intervention Baseline/end SBP (mmHg)	Control base/End SBP (mmHg)	Mean SBP change intervention (mmHg) 95% CI	Between group/effect size (95% CI)	Status	comparator
**Labhardt ND et al [34]**	12 months	175.8/135.6	NR-NR	−40.2(−45.4, −35.0)	NR	Unadjusted	Contract only
**Siedner MJ et al [32]**	12 months	146.6/134.1	147.4/144.8	−10.3 (−12.6, −8.0)		adjusted	Clinic care
**Ogedegbe G et al [33]**	12 months	156/135.6	154.4/137.6	−20.4(−25.2, −15.6)	aMD=-3.6(-6.1, -0.5)	Adjusted	Insurance only
**Oti SO et al [42]**	18 months	161.6/141.6	Single arm/N/A	−19.8 (−23.6, −16.0)	Pre-post only	Unadjusted	None
**Ajay VS et al. [39]**	18 months	146.1/131.5	Single arm/N/A	−14.6 (−15.3 −13.8)	Pre-post only	Unadjusted	None
**Bhattarai S et al [37]**	12 months	134.2/129.8	133.4/130.8	−4.4	aMD=-1.7(-3.4, 0.1)	Adjusted (linear mixed model)	Usual care
**Adler AJ et al [41]**	12 months	147.2/135	Single arm/N/A	−12.2(−14.4, −10.1)	Pre-post only	Unadjusted	None
**Fairall LR et al. [38]**	14 months	134/NR n = 1927	135/NR n = 2048	+1.2	aMD=2.00(-0.87,4.87)	Adjusted	Usual care
**Abrar A et al. [40]**	6 months	158.4/134.7*	158.8/138.8*	−23.7(−24.7, −22.7)	aMD=-3(-5.1, -2.2)	Adjusted	Usual care
**Miao J-H et al. [35]**	4 months	NR	NR	−15.03 ± 23.7	NR	Unadjusted	Usual care

**Footnotes**: None as comparator pre-post within studies; studies with usual care or other comparator report between-group difference, MD = mean difference, aMD = adjusted mean difference, *endline value calculated from baseline and mean reduction of DBP, NR = not reported, *endline value calculated from baseline and mean reduction of SBP

#### Characteristics of diastolic blood pressure (DBP).

Among included studies in our review, nine studies reported DBP outcomes. DBP at baseline ranged from 86.9 mmHg [37] to 100.7 mmHg [34] and endline from 79.8 mmHg [33] to 90.1 mmHg [42] among intervention groups. Whereas baseline DBP ranged from 86.8 [37] mmHg to 92.2 mmHg [40] and endline DBP ranged from 81.8 mmHg [33] to 85.2 mmHg [37] among the control groups. All studies reported a reduction in DBP after the intervention, with a range of −1.8 mmHg [38] to −20.6 mmHg [34] (Table 7).

**Table 7:** Characteristics of diastolic blood pressure control of included studies in review.

Authors	Follow-up	Intervention baseline/End DBP (mmHg)	Control base/End DBP (mmHg)	Intervention mean DBP change (mmHg)	Between group/effect size (95% CI)	Status	comparator
**Ogedegbe G et al [33]**	12 months	89.0/79.8	90.4/81.8	−9.3(−10.5, −8.0)	aMD=-0.6(-2.9, 1.7)	Adjusted for cluster and baseline	Insurance only
**Oti SO et al. [42]**	18 months	100.5/90.1	Single arm	−10.4(−12.7, −8.2)	Pre-post only	Unadjusted	None
**Ajay VS et al. [39]**	18 months	89.52/81.92	Single arm	−7.7(−8.0, −7.3)	Pre-post only	Unadjusted	None
**Adler AJ et al [41]**	12months	89.9/82.4	Single arm	−7.5(−9.9, −6.1)	Pre-post only	adjusted	None
**Abrar A et al. [40]**	6 months	92.3/82.1*	92.2/83.9*	−10.2(−10.8, −9.7)	aMD=-1.9(-1.1, -2.7)	Adjusted baseline	Usual care
**Bhattarai S et al [37]**	12 months	86.9/83.9	86.8/85.2	−3.0	−1.6(−2.6, −0.5)	Adjusted with a linear mixed model	Usual care
**Labhardt ND et al [34]**	12 months	100.7/80.1	NR-NR	−20.6	NR	Unadjusted	Contract only
**Miao J-H et al. [35]**	4 months	NR	NR	−8.54 ± 8.86	NR	Unadjusted	Usual care
**Fairall LR et al. [38]**	14 months	88/NR n = 1927	87/NR n = 2048	−1.8	aMD=1.58(-0.56, 3.72)	Adjusted	Usual care

**Footnotes**: None as comparator pre-post within studies; studies with usual care or other comparator reports between-group difference; MD = mean difference, aMD = adjusted mean difference, *Endline DBP was NR and calculated from reported baseline and mean change after intervention of reported study.

### Effectiveness of interventions

As a result of heterogeneity across the studies, including study design, study follow-up duration, outcome definition, and measurement, a narrative synthesis was employed to evaluate the effectiveness of interventions, following the Synthesis Without Meta-analysis guideline (SWIM) [31]. The narrative synthesis focuses on three outcomes, including retention in care, medication adherence, and blood pressure control (SBP and DBP).

### Retention in care

Across the 11 studies, the current review reflects that studies used different retention measurement approaches, and the follow-up period also varied. Evidence in the current review demonstrated that, while there was wide variability in retention outcome categories, appointment attendance (12.6–96.5%), continuity of care, or routine clinical retention remains sub-optimal (27–62.8%), and study completion of follow-up remains high (79.0% to 97.2%) in primary health care facilities of LMICs. However, study completion of follow-up approach was adherence to study follow-up and may not have merely reflected routine health care follow-up.

Based on review evidence, intervention consistently demonstrated improved retention in care outcomes when clinical protocol-driven follow-up was combined with counseling/education, digital tracking, and health system components. Contextual barriers that were linked with outcomes included transport cost, time constraints, forgetting an appointment, digital system failure due to the crash of the digital platform, cost of care, distance to health care, relocation, adverse drug effects, presence of comorbidity, loss of interest in care, use of alternative medicine, and network problems. Conversely, the presence of telemonitoring and use of SMS reminders, structured clinical follow-up schedule and nurse-led task shifting or task sharing, linked with community health worker, ongoing nurse contact, free medication for care, transport support, home-based visits, improved access, pill box support for daily medication use, and availability of drug supply was identified as a facilitator of patient outcomes. Despite a unified metric for the outcome of retention in care, nurse-led intervention with a combination of proactive clinical follow-up and integration of technology supported improved patients in continuity of care for patients in LMIC settings. In review, none of the studies used integration of nurse-led intervention with one or more than one theory.

### Medication adherence

Out of 11 studies included in the current review, 4 studies [35–37,40] reported medication adherence outcomes and substantial variability in medication adherence measurement across the included studies. Such as pill count, Self-reported medication adherence during follow-up, Satisfactory urine assay test, MMAS-8, and an adherence scoring system, reflecting that the included studies used heterogeneous measurements.

Evidence from the current review shows interventions demonstrated varying effects on medication adherence in the intervention group compared to the control or baseline. Structured medication adherence counselling and education, provision of pill count, integrating reminder systems, and integration of community health care workers for home visits revealed facilitators of medication adherence.

### Blood pressure control

Current review finding shows mean reduction in both SBP and DBP after interventions. Evidence from the current review reports that from 10 reported SBP outcomes, nine reported [32–35,37,39,40,42] mean SBP reduction, and of them, the majority reported the mean reduction of SBP was greater than 10 mmHg [32–35,39–42] after intervention or baseline. Among included studies in our review, nine studies reported DBP outcomes. Even though mean reduction varied across included studies, all studies reported a reduction in DBP after the intervention, with a range of −1.8 mmHg [38] to −20.6 mmHg [34]. The majority of studies reported greater than or equal to 7 mmHg mean DBP reduction after intervention or baseline report, and in particular, studies with utilization of an interlinked, predefined protocol-driven follow-up, integration of technology support, and task-shifting nurse management were predominantly associated with favorable BP reduction. Current review evidence reflects that, in resource-limited settings of LMICs, nurse-led intervention is emerging and promising for blood pressure control with different follow-up window periods.

## Discussion

This systematic review of 11 studies (n = 19,005) demonstrates that nurse-led, multicomponent hypertension care in primary health care settings across LMICs is associated with improvements in blood pressure control. Despite moderately supportive evidence of BP reduction, evidence reported on patient retention was highly heterogeneous, and evidence on medication adherence was limited, with only 4 studies reported.

In the current synthesis, while there is wide variability in retention outcome categories of appointment attendance (12.6–96.5%), continuity of care or routine clinical retention remains sub-optimal (27–62.8%), and study completion of follow-up remains high (79.0% to 97.2%) in primary health care facilities of LMICs. This reflects that hypertension retention lacks a standard operational definition across LMICs. In current studies, retention in care for hypertension was operationalized in distinct methodological approaches, including the number of visit attendance counts with different cutoffs [41], appointment-based [32] and the proportion of scheduled visits attended. This inconsistency of operational definitions creates a significant challenge in cross-study comparison and masks real retention gaps, given the 2024 WHO priority target for retention research [25]. This reflects direct methodological fragmentation and underscores the urgent need for a rigorous yet context-specific operational definition for retention in care. Based on a review of the literature, the hybrid approach was used, and patients were retained if ≥2 visits in 3 months, and each visit occurred within 30 days of the scheduled date [44,45].

The observed change in SBP reduction varied across the studies, and it was a wide range, with a median of −19.8 mmHg within the group. This aligns with prior review on BP outcome indicators for nurse-led interventions in primary health care settings [17]. However, a higher median reduction in our findings might be due to regression to the mean, high baseline blood pressure, medication intensification, temporal improvements in drug availability by the research project rather than sustainable routine health facility capacity, differential attrition, and involvement of a co-intervention.

Clinical effectiveness was observed in the current review with the utilization of a multicomponent nurse-led intervention. Structured counseling and education build knowledge and address misconceptions of illness, while protocol-driven follow-up and digital tracking (SMS, phone calls, mDSS) help cue appointment dates within facilities with weak scheduling systems. Task-shifting to nurses serves as the consistent behavioral anchor, enabling longitudinal relationship-building, trust, and sustained patient engagement. Sustained engagement and medication adherence play a critical role in reducing vascular inflammation, endothelial dysfunction, and vascular smooth muscle phenotypic switching that prevents adverse vascular remodeling and hypertensive end-organ damage [46,47]. This is supported by qualitative and implementation studies conducted across LMICs [11,48,49]. Our synthesis reflects that without deliberate retention strategies, like a theory-driven approach to address both psychosocial and structural barriers, clinical management alone is ineffective for improvement [12].

Contextual barriers and facilitators for included studies. Financial/cost-related barriers, including transport or medicines, access to the facility, patient-level issues like forgetfulness and overdue work, and lack of access to digital support in the health facility, were identified as key barriers across different studies [32,33,36,37,39,41,42]. Contextual factors of healthcare delivery, such as financial and structural shows the broad scope of health setting dynamic whereas variation in resource supply across geographical areas and local health expenditure plays a great role in patients’ sustained engagement in care [50]. Furthermore, physical and living environment are independently determinant for the long-term cardiovascular risk; effectiveness and implementation outcomes should be interpreted within broader environmental and population-level contexts [51]. Free medication provision, transport support, CHW home visits, simplified digital decision support, and structured nurse-led task-shifting were identified as facilitating factors across the studies. This evidence is supported by a study conducted, which reported that traditional education alone is not enough to address the problem [12]. While a study that integrated a clinical protocol-driven approach with a low-cost reminder system and community linkage achieved higher retention and medication adherence, as well as reduced BP control [42]. The effectiveness, acceptability, and sustainability of the intervention depend on when it was designed and backed by theory [52–54].

### Strengths and limitations

The current review has the following methodological strengths: adherence to PRISMA 2020 and use of a multi-database search focusing on LMICs’ primary health care setting; dual independent screening and data extraction minimized selection bias, and our narrative synthesis systematically mapped intervention components, contextual barriers, and facilitators, providing actionable insights for differentiated service delivery.

However, the current study concluded with the following limitations. First, the heterogeneity in study designs and outcome reporting necessitated a narrative synthesis, limiting the ability to draw precise comparative conclusions. Second, heterogeneous outcome definitions, follow-up durations, and measurement tools prevented quantitative pooling and meta-analysis, limiting the precision of our effect estimates. Third, the absence of meta-analysis was due to heterogeneity of outcome measures, different follow-up times, and study designs of the included studies. Fourth, 12 full-text reports could not be retrieved despite inter-library loan requests and direct author outreach. While these unretrieved studies represent potential availability bias, the inclusion of 11 multi-center studies encompassing 19,005 participants provides a representative synthesis of current nurse-led primary care interventions across LMIC settings. Fifth, the current study used three large databases, and database coverage may be limited; CINAHL/Embase were not searched due to resource constraints. Sixth, the included studies were only from LMICs, specifically African and Asian, but lacking study from Latin America may limit geographical generalizability to other LMICs. Seventh, the current study lacks evidence on costs and long-term outcomes. Eighth: retention was rarely designated as a primary outcome in the included trials, suggesting potential underreporting. Finally, the majority of studies had less than or equal to eighteen months of follow-up duration, which may restrict the ability of studies to assess cardiovascular endpoints and long-term effects.

### Implications for Practice and Policy

#### Intervention Effectiveness.

For clinical practice, health systems should prioritize scaling up nurse-led, multi-component interventions in other LMICs with limited access to health care. But to maintain sustainability across different LMIC settings, contextual barriers at the health system level and patient level, such as healthcare workforce capacity and training, timely supervision and close monitoring, sustainable availability of routine health care medicine supply, balanced workload and turnover, digital infrastructure capacity building, and transport costs, need better attention.

### Policy and standardization

Previously, retention in care was considered a policy quality indicator [55]. But in 2024, WHO research on the delivery of hypertension prioritize retention in care listed as one of the research themes and as one of the national non-communicable disease (NCD) monitoring systems [25,56]. Consequently, future studies and policy frameworks should adopt standard operational definitions and measurement tools alongside clinical endpoints such as blood pressure control.

### Implementation feasibility

Interventional studies integrated with theoretical based such as social cognitive theory (SCT), that carefully evaluate and are proven to increase patient self-efficacy, will be needed to overcome any misconceptions in the culture and to ensure sustained engagement. To gain insights regarding the cost-effectiveness and scale of use of digital health tools to achieve robust effectiveness in low-infrastructure contexts, special emphasis must be given to the user-centered development of the system and its integration of the system with the existing health information system.

## Conclusion

This systematic review included 11 studies involving a total of 19,005 participants and showed improvement in blood pressure control by nurse-led interventions in primary health care facilities of LMICs. But retention in care showed substantial variability; routine clinical retention remains sub-optimal (27–62.8%), and study completion of follow-up remains high (79.0% to 97.2%) in nurse-led interventions in primary health care facilities of LMICs. Nurse-led interventions in LMICs are a promising strategy for improving blood pressure control. However, evidence on retention remains inconsistent and of limited certainty due to differences and inconsistencies in measurement and definitions across studies, and evidence on medication adherence was limited. Future research should prioritize standardized outcome measures and robust study designs to strengthen the evidence base and inform sustainable scale-up of these models in LMICs.

## Supporting information

S1 File PRISMA 2020 checklist. (DOCX)

S2 File Search strategy of the review. (DOCX)

S3 File Assessment of risk of bias for included studies. (DOCX)
