# Supplementary material for: Effectiveness of nurse-led hypertension interventions on retention, adherence, and blood pressure control among adults in low- and middle-income countries: A systematic review
Source: PLoS One. 2026 Nov 5;21(11):e0362190. doi: 10.1371/journal.pone.0362190 (PMC13739159; doi:10.1371/journal.pone.0362190)
Supplement: Supplementary file 2 [file pone.0362190.s002.docx]

**S1 File: Search strategy of the review**

**Pubmed/medline searc**h n=336

(("Hypertension"[Mesh] OR hypertension[tiab] OR "high blood pressure"[tiab] OR "antihypertensive agents"[Mesh] OR "Blood Pressure"[Mesh]) AND ("Nurs*"[tiab] OR "Nurse's Role"[Mesh] OR "Nurses"[Mesh] OR "Nursing"[Mesh] OR "Nursing Care"[Mesh] OR "Primary Care Nursing"[Mesh] OR "Community Health Nursing"[Mesh] OR "nurse-led"[tiab] OR "nurse led"[tiab] OR "nurse managed"[tiab] OR "task shifting"[tiab] OR "task sharing"[tiab] OR "non-physician"[tiab] OR "mid-level"[tiab] OR "Clinical Protocols"[Mesh] OR "decision support"[tiab]) AND ("Developing Countries"[Mesh] OR "Africa"[Mesh] OR "Asia"[Mesh] OR "Caribbean Region"[Mesh] OR "Global Health"[Mesh] OR LMIC[tiab] OR LMICs[tiab] OR "low income"[tiab] OR "middle income"[tiab] OR "low and middle income"[tiab] OR "developing countr*"[tiab] OR "third world"[tiab] OR "global south"[tiab] OR "resource limited"[tiab] OR "resource-limited"[tiab] OR "resource poor"[tiab] OR "resource-poor"[tiab] OR "rural population"[Mesh]) AND ( /* Retention terms */ "Patient Dropouts"[Mesh] OR "retention"[tiab] OR "retained"[tiab] OR "retaining"[tiab] OR "attrition"[tiab] OR "dropout"[tiab] OR "drop-out"[tiab] OR "drop outs"[tiab] OR "loss to follow-up"[tiab] OR "lost to follow-up"[tiab] OR "continuity of care"[tiab] OR "care continuity"[tiab] OR "follow-up"[tiab] OR "appointment adherence"[tiab] OR "visit adherence"[tiab] OR /* Adherence terms */ "Medication Adherence"[Mesh] OR "Patient Compliance"[Mesh] OR "adherence"[tiab] OR "complian*"[tiab] OR "non-adherence"[tiab] OR "nonadherence"[tiab] OR "non-complian*"[tiab] OR "persistence"[tiab] OR "medication taking"[tiab] OR "drug adherence"[tiab] OR "treatment adherence"[tiab] OR "pill count"[tiab] OR "medication possession ratio"[tiab] OR "MPR"[tiab] OR "proportion of days covered"[tiab] OR "PDC"[tiab] OR "self-report"[tiab] OR "Morisky"[tiab] OR "MMAS"[tiab] OR "medication refill"[tiab] OR /* Blood pressure control terms */ "blood pressure control"[tiab] OR "BP control"[tiab] OR "controlled hypertension"[tiab] OR "target blood pressure"[tiab] OR "blood pressure goal"[tiab] OR "BP goal"[tiab] OR "blood pressure reduction"[tiab] OR "BP reduction"[tiab] OR "blood pressure lowering"[tiab] OR "antihypertensive treatment"[tiab] OR "blood pressure outcome"[tiab] OR "systolic"[tiab] OR "diastolic"[tiab] OR "mmHg"[tiab] OR "blood pressure measurement"[tiab] ) ) AND ("2000/01/01"[Date - Publication] : "2025/12/15"[Date - Publication])

**Cochrane n=25**

ID	Search	Hits

#1	(Retention in care OR adher* OR continu* OR retain* OR "lost to follow-up" OR "loss to follow-up" OR LTFU OR attrition OR "loss to care" OR "lost to care" OR "loss to program" OR "lost to program" OR "treatment cascade"):ti,ab,kw

#2	("medication adherence" OR "treatment adherence" OR "drug adherence" OR "pill adherence" OR "therapy adherence" OR "pharmacotherapy adherence" OR "prescription adherence" OR "antihypertensive adherence" OR "medication compliance" OR "treatment compliance" OR "drug compliance" OR "Morisky" OR "MMAS" OR "medication possession ratio" OR MPR OR "proportion of days covered" OR PDC OR "pill count" OR "medication refill" OR "adherence rate"):ti,ab,kw

#3	#1 OR #2

#4	("blood pressure" OR systolic OR diastolic OR "bp control" OR "blood pressure control" OR "blood pressure reduction" OR "hypertension control" OR "controlled blood pressure" OR "blood pressure goal" OR "target blood pressure"):ti,ab,kw

#5	#3 OR #4

#6	(hypertension OR "high blood pressure"):ti,ab,kw

#7	("primary care" OR "primary health care" OR "primary healthcare" OR "community health" OR "community care" OR "rural health"):ti,ab,kw

#8	(nurse-led OR nurse-managed OR "nurse directed" OR "nurse driven" OR "nurse based" OR "nurse coordinated" OR "task sharing" OR "task-shifting" OR "delegated care" OR "protocol based titration" OR "defaulter tracing" OR "structured follow-up" "family nurse" OR "primary nursing" OR "visiting nursing service" OR "nurse practitioner" OR "registered nurse" OR "nurse clinician" OR "community nurse" ):ti,ab,kw

#9	(lmics OR "low income" OR "middle income" OR "developing countr*" OR "global south" OR africa OR asia OR "latin america "):ti,ab,kw

#10	#9 AND #7 AND #6 AND #5 AND #8

**Scopus** n=142

( TITLE-ABS-KEY ( "hypertension" OR "high blood pressure" OR "blood pressure control" ) AND TITLE-ABS-KEY ( "nurse-led" OR "nurse managed" OR "nurse delivered" OR "nursing intervention" OR "task shifting" OR "task sharing" OR "non-physician-led" OR "team-based care" OR "community health worker" ) AND TITLE-ABS-KEY ( "primary health care" OR "primary healthcare" OR "health center" OR "community health" OR "primary care" ) AND TITLE-ABS-KEY ( "intervention" OR "program" OR "care model" OR "management model" OR "implementation" ) AND TITLE-ABS-KEY ( "Africa" OR "low- and middle-income countries" OR "LMIC" OR "developing countries" ) ) AND PUBYEAR > 1999 AND PUBYEAR < 2027
