# Supplementary material for: Effectiveness of nurse-led hypertension interventions on retention, adherence, and blood pressure control among adults in low- and middle-income countries: A systematic review
Source: PLoS One. 2026 Nov 5;21(11):e0362190. doi: 10.1371/journal.pone.0362190 (PMC13739159; doi:10.1371/journal.pone.0362190)
Supplement: Supplementary file 3 [file pone.0362190.s003.docx]

**S1 File:** Assessment of risk of bias for included studies: Comprehensive domain-level risk of bias evaluation using Cochrane RoB 2 for randomized controlled trials and JBI critical appraisal tools for quasi-experimental and cohort studies.

1. The Cochrane Risk of Bias Tool was used to assess the risk of bias for RCTs included in the reviews

| study | D1 | D2 | D3 | D4 | D5 | Overall |
| --- | --- | --- | --- | --- | --- | --- |
| Labhardt N et al 2011 | some concern | Low risk | High risk | Low risk | some concern | High risk |
| Miao JH et al. 2020. | some concern | Low risk | High risk | Low risk | some concern | High risk |
| Bhattarai S et al. 2024 | some concern | Low risk | High risk | Low risk | Low risk | Some concern |
| Adeyemo A et al. 2013 | some concern | High risk | High risk | Low risk | some concern | High risk |
| Siedner MJ et al. 2025 | Low risk | Low risk | Low risk | Low risk | Low risk | Low risk |
| Ogedagbe et al.2018 | Low risk | Low risk | Low risk | Low risk | Low risk | Low risk |
| Fairall LR et al. 2016 | some concern | some concern | Low risk | Low risk | Low risk | some concern |

Domain D1=Bias raising from randomization,

D2 = Bias due to deviation from the intended intervention

D3 =Bias due to missing outcome data

D4 =Bias due to measurement of outcome

D5 = Bias due to the selection of the reported result

2. Joanna Briggs Institute critical appraisal checklist for quasi-experimental studies; quality and risk of bias of included studies

| Component of assessment | Ajay VS et al. 2016 | Ahmad Abra et al.2024 |
| --- | --- | --- |
| Clear cause & effect | Yes | Yes |
| Participants included in any comparisons similar | No | No |
| Participants included in any comparisons received similar care | No | No |
| Control group | No | No |
| Multiple measures of outcome, both pre/post-intervention | Yes | Yes |
| Complete follow-up, any differences described & analyzed | Yes | Yes |
| Outcomes of participants recorded in same way | Not applicable | Not applicable |
| Outcomes reliably measured | Yes | Yes |
| Appropriate statistical analysis used | Yes | Yes |
| Total | 5 | 5 |
| Overall risk of bias | 62.5% | 62.5% |
| Overall risk of bias grade | Moderate risk | Moderate risk |

3. Joanna Briggs Institute critical appraisal checklist for cohort study; quality and risk of bias of included studies

| Component of assessment | Adler et al., 2019 | Oti et al. 2016 |
| --- | --- | --- |
| Two groups were similar and recruited from the same population | No | Yes |
| Exposures were measured similarly to assign people to both the exposed and unexposed groups | Not applicable | Not applicable |
| Exposure measured in a valid and reliable way | Yes | Yes |
| Confounding factors identified | Yes | Yes |
| Strategies to deal with confounding factors stated | Yes | Yes |
| groups/participants free of the outcome at the start of the study (or at the moment of exposure | Yes | Yes |
| Outcomes measured in a valid and reliable way | Unclear | Unclear |
| Follow-up time reported and sufficient to be long enough for outcomes to occur | Yes | Yes |
| Follow-up complete, and if not, were the reasons for the loss to follow-up described and explored | Yes | Yes |
| Strategies to address incomplete follow-up utilized | No | No |
| An appropriate statistical analysis was used | Yes | Yes |
| Total | 7 | 7 |
| Overall risk of bias | 70% | 70% |
| Overall risk of bias grade | Low risk | Low risk |

**Figure 2a**: Risk of bias for randomized control trails (Cochrane RoB2 risk of bias assessment) included in the studies

**Figure 2b:** Risk of bias for quasi-experimental trials (JBI Tool) included in the studies

**Figure 2c**: Risk of bias for cohort studies included by the JBI tool
